# Lipidic Aminoglycoside Derivatives: A New Class of Immunomodulators Inducing a Potent Innate Immune Stimulation

**DOI:** 10.1002/advs.201900288

**Published:** 2019-07-15

**Authors:** Thibault Colombani, Thomas Haudebourg, Marion Decossas, Olivier Lambert, Grace Ada Da Silva, Frederic Altare, Bruno Pitard

**Affiliations:** ^1^ CRCINA, INSERM Université d'Angers, Université de Nantes Boulevard Bénoni Goullin Nantes 44200 France; ^2^ CBMN UMR‐CNRS 5248 Université de Bordeaux Allée Geoffroy Saint Hilaire Pessac 33600 France

**Keywords:** adjuvants, cationic lipids, immunotherapy, lipoaminoglycosides, phosphatidylinositol phospholipase C (PLC)

## Abstract

Development of simple and fully characterized immunomodulatory molecules is an active area of research to enhance current immunotherapies. Monophosphoryl lipid A (MPL), a nontoxic lipidic derivative from bacteria, is the first and currently only adjuvant approved in humans. However, its capacity to induce a potent response against weak immunogenic tumoral‐associated antigens remains limited. Herein, a new generation of lipidic immunomodulators to conduct a structure–activity relationship study to determine the minimal structural elements conferring immunomodulatory properties is introduced. Two lead molecules characterized by a short succinyl linker between two oleyl chains and a polar headgroup consisting of either naturally occurring tobramycin (DOST) or kanamycin (DOSK) are identified. These two lipoaminoglycosides self‐assemble in very small vesicles. In a wide variety of cells including 3D human cell culture, DOST and DOSK induce the upregulation of proinflammatory cytokines and interferon‐inducible proteins in a dose and time‐dependent manner via a caveolae‐dependent proinflammatory mechanism and phosphatidylinositol phospholipase C activation. Furthermore, after intratumoral administration, these lipoaminoglycosides induce an efficient immune response leading to significant antitumor activity in a mouse breast cancer model. Altogether, these findings indicate that DOST and DOSK are two groundbreaking synthetic lipid immunostimulators that can be used as adjuvants to enhance current immunotherapeutic treatments.

## 1 Introduction

The immunosuppressive tumor environment has recently been the focus of much research aimed at preventing tumor cells from evading immune attack. This lead to the emergence of checkpoint inhibitors, including antiprogrammed death protein 1 (PD‐1) or anti‐PD ligand 1 (PD‐L1) antibodies, that block immunosuppressive pathways and allow the host immune system to resume its ability to recognize and kill tumor cells.^1^ However, these anti‐PD1 inhibitors are only effective in a relatively small proportion of patients when used alone because checkpoint blockage can only work if an adequate antitumor immune response has been developed.^2^ There is, therefore, a strong rationale to combine checkpoint blockades with an effective immunostimulation that specifically induces antitumor activities. However, to date, the use of vaccine strategies has often failed because of poor efficacy in stimulating immune cells with sufficient functionality to kill tumor cells. Indeed, these classes of vaccines are usually weakly immunogenic and require the use of adjuvants to induce strong and long‐lasting anticancer immune responses.^3^

Recently, the development of liposome‐based adjuvants has gained momentum. Their lipidic composition, charge, and size may be easily modified, making them extremely versatile tools tunable for each specific applications.^4^ Monophosphoryl lipid A (MPL), a chemically detoxified derivative of bacterial lipopolysaccharide (LPS),^5^ is a typical example. MPL liposomes are potent activators of dendritic cells (DCs), T cells, and B cells, and are already used as adjuvants in licensed vaccines in combination with antigens or aluminum salts.^6^, ^7^ MPL liposomes can bind to toll‐like receptor (TLR) 4,^8^ increasing the production of proinflammatory cytokines and chemokines^9^ through the activation of the NFκB pathway.^10^ However, improving the activation of the immune system is still an active and challenging area of research. Indeed, the study of live‐attenuated vaccines has highlighted the crucial importance of activating multiple signaling pathways relative to innate immunity.^11^ Current adjuvants often target a single receptor (e.g., TLR) or signaling pathway (e.g., NFkB) leading to incomplete immune stimulation. Thus, discovering a lipidic molecule able to both stimulate NFkB‐dependent and ‐independent signaling pathways while interacting with multiple receptors could potentially be of great interest to maximize innate immune stimulation.

Aminoglycosides, a family of amino‐modified sugars produced by fungi, are capable of stimulating acute renal cell signaling^12^ by allosterically activating phosphatidylinositol phospholipase C (PLC).^13^ This results in an increase in intracellular calcium levels and activation of the extracellular signal‐regulated kinase (ERK) pathway in a dose‐ and time‐dependent manner,^14^ leading ultimately to a proinflammatory response.^15^ Thus, we hypothesized that lipidic derivatives of naturally occurring aminoglycosides could activate both NFkB‐dependent and ‐independent pathways (e.g., PLC–ERK), paving the way for breakthrough mechanisms of innate stimulation.

To address this hypothesis, we have first conducted a structure–activity relationship study based on cationic lipid compounds previously prepared for structural optimization programs intended for intracellular delivery of macromolecules.^16^, ^17^, ^18^, ^19^, ^20^ Fourteen different lipoaminoglycosides bearing different aminoglycoside polar headgroups (paromomycin, neomycin B, kanamycin A, tobramycin), spacers (succinyl, adipyl, serinyl, dithioglycolyl), or hydrophobic segments (dioleyl, distearyl, dipalmityl, dimyristyl, cholesterol) have been examined to determine the structural elements necessary to confer them immunomodulatory properties. This allowed the identification of two synthetic molecules (namely, tobramycin (DOST) and kanamycin (DOSK)), which are able to strongly stimulate the cell immunity in vitro in a wide panel of cells, including 3D‐organized human cells. In a second step, we investigated the different signaling pathways and endocytosis mechanisms involved in innate immune stimulation. Finally, we tested the ability of the lipoaminoglycosides to enhance antitumor activity. Indeed, DOSK decreased tumor growth in a highly relevant preclinical 4T1 mouse model of breast cancer,^21^ confirming the immunostimulatory properties of this new class of liposome‐based adjuvants.

## 2 Results

### 2.1 Impact of Lipoaminoglycoside Structure on Innate Immune Stimulation

**Figure 1:**
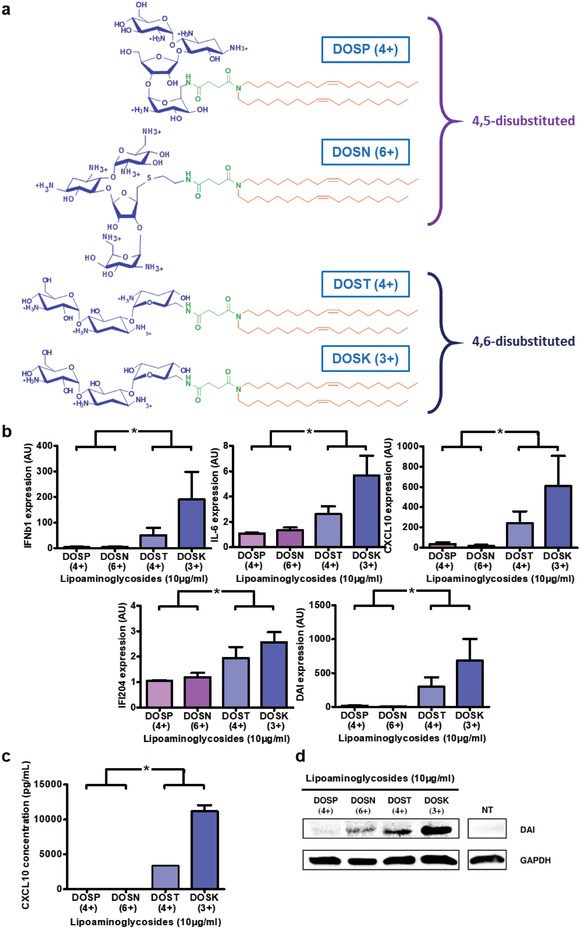
4,6‐Disubstituted polar heads group kanamycin and tobramycin allow the best immune stimulation. a) Structure of di‐oleyl‐succinyl derived cationic lipids: DOSP (di‐oleyl‐succinyl‐paromomycin), DOSN (di‐oleyl‐succinyl‐neomycin B), DOST (di‐oleyl‐succinyl‐tobramycin), and DOSK (di‐oleyl‐succinyl‐kanamycin A). b–d) Stimulation of innate immune response by di‐oleyl‐succinyl derived cationic lipids in C2C12 cells. Mouse myoblast cells (*n* = 4) were incubated for 24 h with 10 µg mL^−1^ of DOSP, DOSN, DOST or DOSK, belonging to the 4,5‐disubstituted 2‐deoxystreptamine ring subclass or 4,6‐disubstituted 2‐deoxystreptamine ring subclass respectively. b) Cytokine (IFNβ1 and IL‐6), chemokine (CXCL10), and DNA sensor (DAI and IFI204) expression levels in cells were determined by RT‐qPCR analysis, normalized against the expression levels of HPRT (housekeeping gene) and compared to nontreated cells (AU = 1). Data are expressed as the 2‐∆∆CT formula. c) Chemokine CXCL10 level into supernatant was determined by ELISA analysis. d) DAI protein production by cells was determined by Western Blot analysis and the level of GAPDH was used as a protein loading control. Values are shown as mean ± SEM. Data were analyzed using Mann–Whitney test, *p < 0.05.

To characterize the influence of aminoglycoside polar headgroups on innate immune stimulation, we prepared four molecules carrying the same hydrophobic segment (dioleyl chains) and spacer (succinyl) but different aminoglycoside headgroups: DOSP (paromomycin headgroup, four protonatable amines), DOSN (neomycin B headgroup, six protonatable amines), DOST (tobramycin headgroup, four protonatable amines), and DOSK (kanamycin A headgroup, three protonatable amines). The structures of the different molecules are depicted in **Figure** **1**a. These four compounds can be divided into two subclasses, as paromomycin and neomycin are characterized by a 4,5‐disubstituted 2‐deoxystreptamine (4,5‐DDS) ring, while tobramycin and kanamycin are characterized by a 4,6‐disubstituted 2‐deoxystreptamine (4,6‐DDS) ring.

The immunomodulatory properties of these lipoaminoglycosides were investigated using C2C12 cells as an in vitro model due to their conservation of an intact NFkB and interferon response system.^22^ Activation of the NFkB‐dependent pathway has been monitored by following interleukin 6 (IL‐6) and chemokine (C‐X‐C motif) ligand 10 (CXCL10) expression and production. NFkB‐independent pathway stimulation has been investigated by quantifying the expression and production of interferon β1 (IFNβ1), as well as DNA‐dependent activator of interferon regulatory factors (DAI) and interferon‐gamma induced protein 204 (IFI204), well known as DNA sensors. Preliminary studies using DOSP (Figure S1a, Supporting Information) to determine the optimal concentration and treatment duration showed that at least 10 µg mL^−1^ of lipoaminoglycosides (Figure S1b, Supporting Information) for 24 h (Figure S1c, Supporting Information) was required to trigger a significant cell stimulation. Therefore, these parameters have been used for the full structure–activity study. The treatment of C2C12 cells with DOSP or DOSN only led to a slight overexpression of IFNβ1, IL‐6, CXCL10, DAI, and IFI204 compared to untreated cells (Figure 1b). By contrast, DOST and DOSK treatment enhanced by 50‐ to 500‐fold the expression of IFNβ1, CXCL10, and DAI, and most importantly, they induced a significant increase of IL‐6 and IFI204 expression. ELISA analysis of CXCL10 secretion in the supernatant induced by DOST and DOSK was consistent with the gene expression results (Figure 1c), unlike what was observed with DOSP and DOSN treatments. Similarly, western blotting assay also demonstrated that DOST and DOSK induced a higher level of DAI protein production within C2C12 cells (Figure 1d) compared to DOSP and DOSN treatments.

The ability of polar headgroups alone (i.e., aminoglycosides not linked to any hydrophobic moiety) to stimulate cellular immunity was also analyzed (Figure S2, Supporting Information). Treatment of C2C12 cells with paromomycin, neomycin B, tobramycin, or kanamycin A did not lead to any cell stimulation, suggesting the key implication of the amphiphilic character of lipoaminoglycosides to trigger immune stimulation. To confirm this hypothesis, we synthesized four new amphiphilic aminoglycoside derivatives (CHOLP, CHOLN, CHOLT, CHOLK) having the same polar headgroup but a cholesterol hydrophobic moiety (Figure S3a, Supporting Information). Surprisingly, C2C12 cells treated with these cholesterol derivatives did not show any increase of both immune genes expression (Figure S3b, Supporting Information) or DAI protein production (Figure S3c, Supporting Information), regardless of the polar head group composition. Therefore, an amphiphilic structure and presence of a diacyl hydrophobic segment seem to be crucial to confer immunomodulatory properties on lipoaminoglycosides.

**Figure 2:**
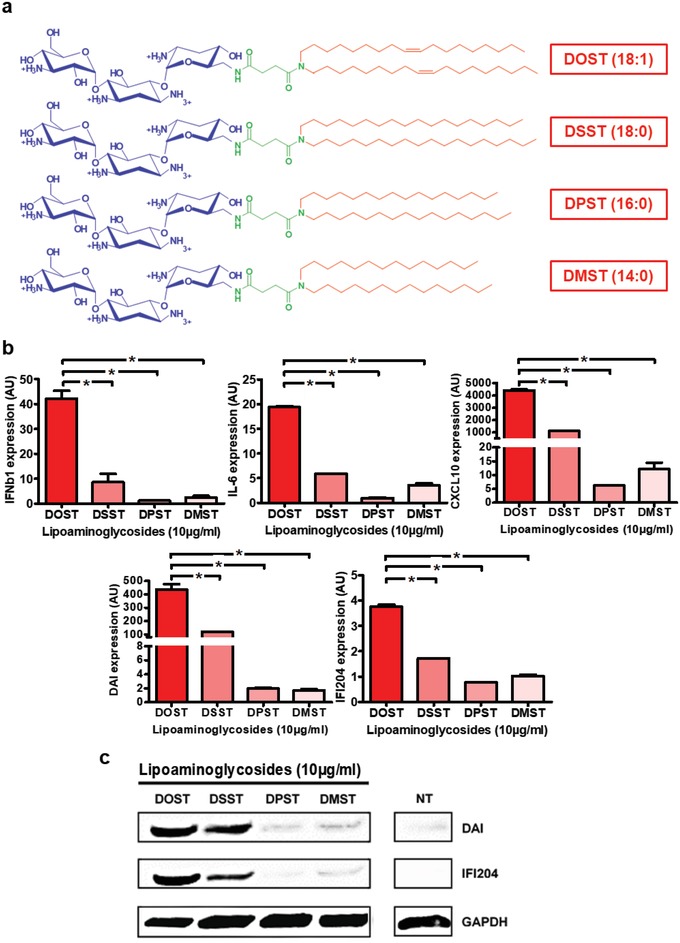
Implication of hydrophobic segment on C2C12 immune stimulation. a) Structure of tobramycin derived cationic lipids. DOST (di‐oleyl‐succinyl‐tobramycin), DSST (di‐stearyl‐succinyl‐tobramycin), DPST (di‐palmityl‐succinyl‐tobramycin), and DMST (di‐myristyl‐succinyl‐tobramycin). b,c) Stimulation of innate immune response by tobramycin derived cationic lipids in C2C12 cells. Mouse myoblast cells (*n* = 4) were incubated for 24 h with 10 µg mL^−1^ of DOST, DSST, DPST, or DMST. b) Cytokine (IFNβ1 and IL‐6) and chemokine (CXCL10) and DNA sensor (DAI and IFI204) expression levels in cells were determined by RT‐qPCR analysis, normalized against the expression levels of HPRT (housekeeping gene) and compared to nontreated cells (AU = 1). Data are expressed as the 2‐∆∆CT formula. c) DNA sensor (DAI and IFI204) protein levels were determined by Western Blot analysis and the level of GAPDH was used as a protein loading control. Values are shown as mean ± SEM. Data were analyzed using Mann–Whitney test, *p < 0.05.

Thus, we next investigated the influence of the diacyl hydrophobic segment on C2C12 cell stimulation. Starting from DOST, which possesses a tobramycin headgroup, a succinyl spacer, and a hydrophobic dioleyl chain (oleyl: 18 carbons with 1 unsaturation, C18:1), we synthesized three new molecules having different hydrophobic tails: (i) DSST with a distearyl chain (stearyl: saturated 18 carbons, C18:0), (ii) DPST with a dipalmityl chain (palmityl: saturated 16 carbons, C16:0), and (iii) DMST with a dimyristyl chain (myristyl: saturated 14 carbons, C14:0) (**Figure** **2**a). Removing the unsaturation (DSST treatment) led to a dramatic decrease of C2C12 immune stimulation by two‐ to fourfold (Figure 2b) compared to DOST. Similarly, shortening the saturated alkyl chains (DMST and DPST treatments) also led to a much lower expression of IFNβ1, IL‐6, and CXCL‐10, or even to no effect on DAI and IFI204 expression compared to untreated cells. The analysis of DAI and IFI204 protein production by C2C12 cells further confirmed these results, as only DOST and DSST treatments induced an increased protein production (Figure 2c).

**Figure 3:**
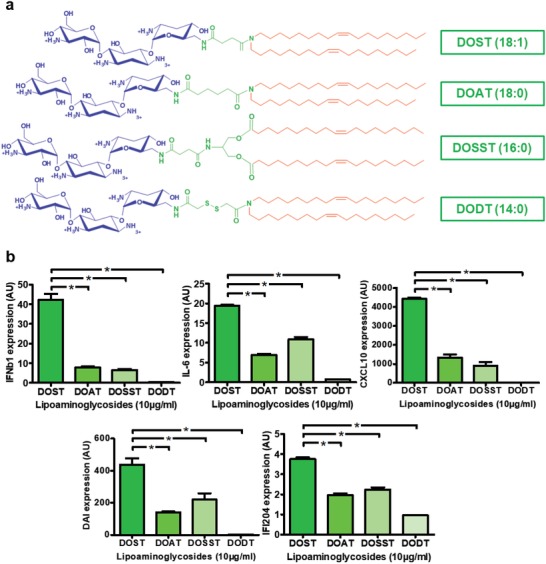
Importance of linker composition on C2C12 immune stimulation. a) Structure of di‐oleyl‐tobramycin derived cationic lipids DOST (di‐oleyl‐succinyl‐tobramycin), DOAT (di‐oleyl‐adipyl‐tobramycin), DOSST (di‐oleyl‐succinyl‐serinyl‐tobramycin), and DODT (di‐oleyl‐dithioglycolyl‐tobramycin). b,c) Stimulation of innate immune response by di‐oleyl‐tobramycin derived cationic lipids in C2C12 cells. Mouse myoblast cells (*n* = 4) were incubated for 24 h with 10 µg mL^−1^ of DOST, DOAT, DOSST or DODT. b) Cytokine (IFNβ1 and IL‐6), chemokine (CXCL10), and DNA sensor (DAI and IFI204) expression levels in cells were determined by RT‐qPCR analysis, normalized against the expression levels of HPRT (housekeeping gene) and compared to nontreated cells (AU = 1). Data are expressed as the 2‐∆∆CT formula. Values are shown as mean ± SEM. Data were analyzed using Mann–Whitney test, *p < 0.05.

Finally, we determined the influence of the spacer linking the aminoglycoside headgroup with the hydrophobic moiety on innate immune stimulation. From DOST, our reference molecule bearing a “succinic” spacer, three new lipoaminoglycosides were synthesized by (i) adding two methylene units forming an “adipic” spacer (DOAT), (ii) introducing a diester function (DOSST), or (iii) inserting a disulfide bridge (DODT) (**Figure** **3**a). As shown in Figure 3b, C2C12 cells treated with DOAT and DOSST had a lower expression of DAI (twofold decrease), IFI204 (twofold), IL‐6 (threefold), IFNβ1 (fourfold), and CXCL10 (fourfold) compared to DOST treatment. Interestingly, the DODT compound with the disulfide bridge was unable to induce any cell stimulation.

Based on these data, we selected the top‐two molecules, DOST and DOSK, for further evaluation. To demonstrate their universal adjuvant properties, we assessed their ability to stimulate the immune response of a wide panel of cell populations (Figure S4, Supporting Information). As expected, both DOST and DOSK were able to induce a strong and efficient innate immune stimulation of mouse embryonic fibroblasts (MEFs), murine immature dendritic cells (JAWSII), primary peritoneal cavity cells, and bone‐marrow‐derived dendritic cells (BMDCs).

### 2.2 The Immune Stimulation Induced by Lipoaminoglycosides Is Dependent on Caveolae‐Mediated Endocytosis

**Figure 4:**
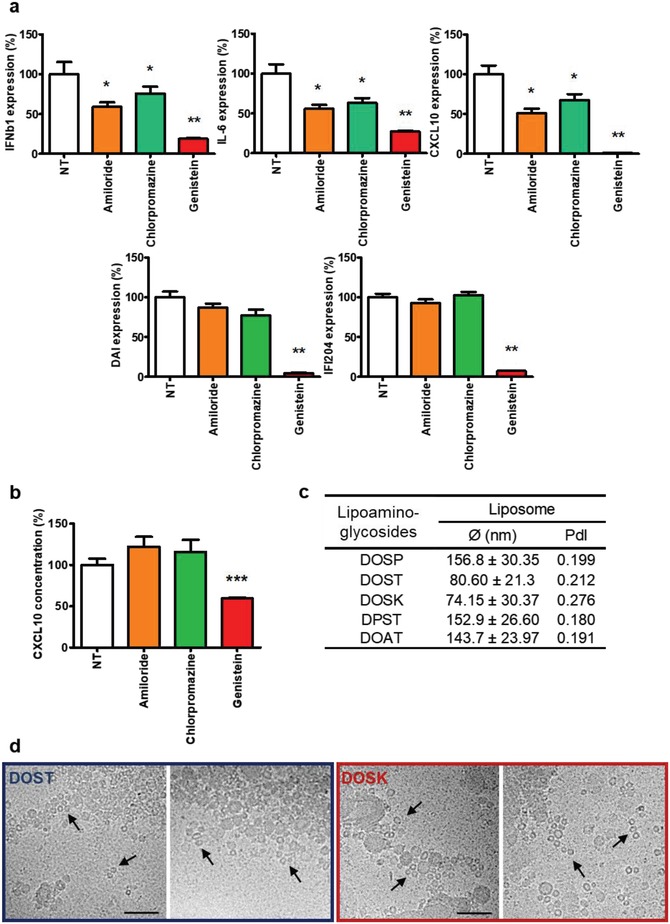
Role of endocytosis in C2C12 immune genes expression induced by DOSK. a,b) Mouse myoblast cells (*n* = 6) were incubated for 24 h with DOSK at a concentration of 10 µg mL^−1^ in the presence or absence of 150 × 10^−9^ m of amiloride or 9 × 10^−6^ m of chlorpromazine or 90 × 10^−6^ m of genistein. a) Cytokine (IFNβ1 and IL‐6), chemokine (CXCL10), and DNA sensor (DAI and IFI204) expression levels in cells were determined by RT‐qPCR analysis, normalized against the expression levels of HPRT (housekeeping gene) and compared to cells treated without inhibitors (expression = 100%). Data are expressed as the 2‐∆∆CT formula. b) Chemokine CXCL10 level into supernatant were determined by ELISA analysis and compared to cells treated without inhibitors (concentration = 100%). c) Values of the diameters and PdI of different aminoglycoside lipidic derivatives (DOSP, DOST, DOSK, DPST, and DOAT) formulated at 10 µg mL^−1^ estimated by dynamic light scattering. d) CryoEM pictures of DOST and DOSK in complete DMEM medium. The presence of very small unilamellar vesicles with 15 nm in diameter is indicated (black arrows). Scale bar 100 nm. Values are shown as mean ± SEM. Data were analyzed using Mann–Whitney test, *p < 0.05, **p < 0.01, and ***p < 0.01.

Lipoaminoglycosides belong to the ionizable lipids family, and it is well admitted that electrostatic interaction between ionizable lipids and cell membranes results in their internalization via endocytosis.^23^ Therefore, we investigated the implication of endocytosis mechanisms on the ability of lipoaminoglycosides to stimulate cell immune response. To this aim, C2C12 cells were treated for 24 h with 10 µg mL^−1^ of DOSK, in the presence or absence of endocytosis inhibitors: (i) amiloride (a macropinocytosis inhibitor), (ii) chlorpromazine (a clathrin‐mediated endocytosis inhibitor), or (iii) genistein (a caveolae‐mediated endocytosis inhibitor). These inhibitors were first assessed for both their toxicity (Figure S5a, Supporting Information) and efficacy using known control compounds (Figure S5b, Supporting Information) to determine the working concentration. Although amiloride and chlorpromazine treatments inhibited IFNβ1, IL‐6, and CXCL10 expression by 30% to 50% compared to nontreated cells (**Figure** **4**a), they had no impact on DAI and IFI204 overexpression induced by DOSK. On the contrary, inhibiting caveolae‐mediated endocytosis using genistein treatment not only dramatically inhibited IFNβ1, Il‐6, and CXCL10 expression (70–95% reduction compared to nontreated cells), but also drastically reduced DAI and IFI204 expression. Similar results were obtained by analyzing CXCL10 secretion by C2C12 cells, as only genistein treatment resulted in a decrease of CXCL10 concentration in the supernatant of DOSK‐treated cells compared to nontreated ones (Figure 4b).

Caveolin‐mediated endocytosis is mainly used by mammalian cells to internalize particles measuring 20 to 100 nm.^24^, ^25^ Thus, we characterized the size of different lipoaminoglycosides used in this study to determine the correlation between their size, endocytosis mechanism, and immunostimulatory properties. Dynamic light scattering (DLS) analysis showed that only the immunostimulatory molecules DOST and DOSK formed small vesicles (80.6 ± 21.3 and 74.15 ± 30.37 nm, respectively) compared to DOSP, DPST, and DOAT (above 140 nm) (Figure 4c). Furthermore, cryoEM images of DOST and DOSK showed that in addition of their ≈80 nm multilamellar structures, they also exhibited many small unilamellar vesicles with diameters of 13.6 ± 3.7 and 12.2 ± 4.8 nm, respectively (Figure 4d – black arrows). Taken together, these data support that the endocytosis of lipoaminoglycosides is a prerequisite for the induction of an efficient innate immune stimulation. Moreover, the caveolae‐mediated endocytosis appeared to be the most efficient pathway, likely due to the small vesicular structures of these lipidic adjuvants.

### 2.3 Immunostimulatory Properties of DOST and DOSK Is TLR2‐ and TLR4‐Independent but PLC‐Dependent

**Figure 5:**
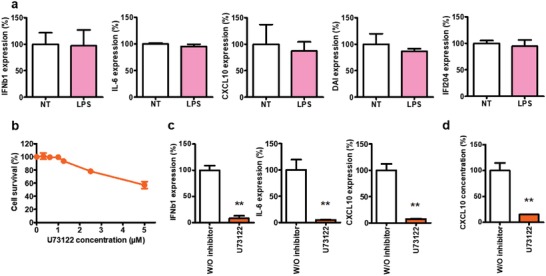
DOSK activates cell immunity through PLC‐dependent signaling pathway. a) Mouse myoblast cells (*n* = 6) were incubated for 24 h with or without 10 µg mL^−1^ of LPS. Cytokine (IFNβ1 and IL‐6) and chemokine (CXCL10) expression levels in cells were determined by RT‐qPCR analysis, normalized against the expression levels of HPRT (housekeeping gene) and compared to cells treated without LPS (expression = 100%). b) Percentage cell survival of C2C12 cells. Mouse myoblast cells (*n* = 3) were treated with U73122 at a concentration of 0 to 5 × 10^−6^ m during 24 h. Cell viability was evaluated using the Cell Titer 96 nonradioactive cell proliferation assay. c,d) Mouse myoblast cells (*n* = 6) were incubated for 24 h with DOSK at a concentration of 10 µg mL^−1^ in presence or absence of 1 × 10^−6^ m of U73122. c) Cytokines (IFNβ1 and IL‐6) and chemokine (CXCL10) expression levels into cells were determined by RT‐qPCR analysis, normalized against the expression levels of HPRT (housekeeping gene) and compared to cells treated without inhibitors (expression = 100%). d) Chemokine CXCL10 level into supernatant was determined by ELISA analysis and compared to cells treated without inhibitors (concentration = 100%). Values are shown as mean ± SEM. Data were analyzed using Mann–Whitney test, *p < 0.05, **p < 0.01.

TLR2 and TLR4 are extensively described as the main receptors involved in the recognition of ionizable lipid hydrophobic segments.^26^, ^27^, ^28^ However, for our study, we used C2C12 cells which lack TLR2 receptors^29^ and fail to show any functional TLR4‐mediated signalization, as exemplified by the absence of LPS' effect on cell stimulation (**Figure** **5**a). Using HEK293 cells (Figure S6, Supporting Information), we further confirmed that neither the expression of the human TLR2 receptor (hTLR2) nor the human TLR4 receptor (hTLR4) changed the expression of IL‐6, DAI, and IFI204 after DOST or DOSK treatment compared to HEK293 cells lacking both TLR2 and 4 (null–negative control). More strikingly, the expression of TLR2 and TLR4 drastically inhibited IFNb1 and CXCL10 expression after lipoaminoglycoside treatments. These results strongly suggest that the immunostimulatory properties of DOST, DOSK, and the other lipoaminoglycosides observed in our work were very likely TLR2‐ and TLR4‐independent.

We next evaluated the ability of lipoaminoglycosides to stimulate the PLC signaling pathway. C2C12 cells were treated with DOSK in the presence or the absence of 1 × 10^−6^ m of the PLC inhibitor U73122, a working concentration that did not induce any cell toxicity (Figure 5b). Under this condition, the inhibition of PLC led to a dramatic reduction of IFNβ1, IL‐6, CXCL10, and DAI expression (Figure 5c), as well as CXCL10 secretion in cell supernatant (Figure 5d).

### 2.4 Validation of Lipoaminoglycoside Immunomodulatory Efficacy

**Figure 6:**
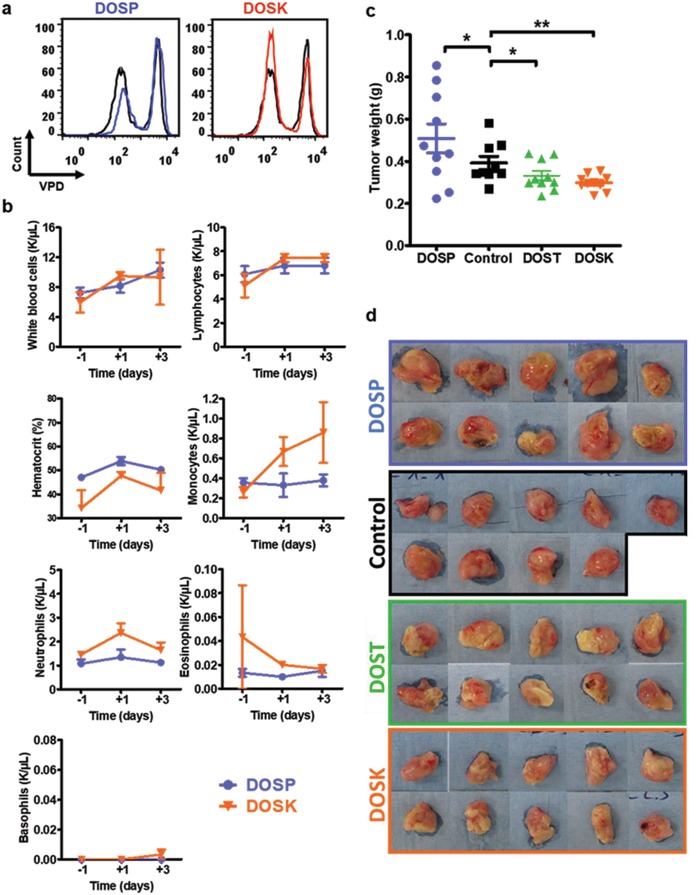
Ex vivo and in vivo validation of lipoaminoglycosides immunostimulatory properties. a) Ex vivo proliferative responses of granuloma human T cells to bacille calmette‐guerin (BCG) in the presence of 10 µg mL^−1^ of DOSP and DOSK. Human PBMCs were labeled with violet proliferation dye (VPD) and cultured in the presence of BCG at an MOI of 1 for 7 days. Black histograms correspond to proliferation of granulomas T cells in response to BCG (gated on T cells) and blue and red histograms correspond to proliferation of granulomas T cells cocultured with BCG and 10 µg mL^−1^ of the indicated compound. The VPD dilution was analyzed by flow cytometry and the Flowlogic software. Results from one representative experiment out of three are shown. b) In vivo toxicity. Swiss mice (*n* = 3) were injected intravenously at day 0 with 100 µg of DOSP or DOSK. Blood formulation and hematocrit level of each mouse were monitored from 1 day before and 3 days after injection. c,d) BALB/c mice bearing orthotopic 4T1 tumors were treated with two weekly intratumoral injections of control vehicle, DOSP, DOST or DOSK beginning on day 10 after intramammary injection of tumor cells (*n* = 10 per group). At day 19 after intramammary injections, tumors were excised and weighed (c), then photographed (d). One mouse in the control group died during the experiment. Values are shown as mean ± SEM. Data were analyzed using Mann‐Whitney test, *p < 0.05, **p < 0.01.

To demonstrate the immune‐modulatory properties of lipoaminoglycosides in more complex models, we first used a granuloma model consisting of human cells organized in a 3D structure. This in vitro model mimics the complex immune reaction occurring between human peripheral blood cells and mycobacteria as previously described.^30^ Although DOSP did not induce any change in granulomatous T cell proliferation compared to control experiments (black lines, granulomas in the absence of lipoaminoglycosides), DOSK treatment led to an increase in T cell stimulation and proliferation within the granuloma, confirming its immunostimulatory potency (**Figure** **6**a).

Next, we investigated the immunomodulatory potency of lipoaminoglycosides in vivo using mice. To ensure the absence of toxicity relative to lipoaminoglycoside administration, Swiss mice were injected intravenously with 100 µg of DOSP or DOSK in 100 µL of phosphate‐buffered saline (PBS), and their blood composition, as well as hematocrit levels, were monitored from 1 day before and up to 3 days after injection (Figure 6b). Results show that neither DOSP nor DOSK injection induced a significant variation in white blood cells, lymphocytes, neutrophils, eosinophils, and basophils concentration compared to the pretreatment day. Similarly, there were no observed impacts on hematocrit levels from DOSP or DOSK injection. Although a slight increase in term of monocyte concentration has been measured using DOSK, the values obtained remained within the normal physiological range (0.3–1.4 K µL^−1^). Then, we examined the capability of DOSP, DOST, and DOSK to impact tumor growth in a well‐established BALB/c 4T1 orthotopic breast tumor model.^31^ Starting 10 days after intramammary injection of tumor cells (tumor volume = 200 mm^3^), mice were treated with two weekly intratumoral injections of either saline solution (control), 1 mg of DOSP, 1 mg of DOST or 1 mg of DOSK. At day 19, tumors were excised, weighed and photographed. As expected, tumors treated with DOST and DOSK had their growth significantly inhibited resulting in reduced tumor weight (Figure 6c) and size (Figure 6d) compared to the control treatment. By contrast, DOSP treatment did not lead to tumor regression. Altogether, these results strongly suggest that DOST and DOSK intratumoral injection was able to enhance the immune response locally, resulting in decreased 4T1 mammary tumor growth.

## 3 Discussion

A key challenge in cancer immunotherapy is to increase the percentage of patients responsive to treatments. Indeed, current strategies have poor clinical outcomes, with only subsets of patients sensitive to therapies alone. This could be overcome by combining current cancer immunotherapies with intratumoral administration of more potent immunostimulators that can trigger the multiple facets of the immune response, without inducing systemic inflammatory toxicity. Here, the data set provides key structural parameters to consider while developing lipid‐based immunostimulators. Moreover, we have demonstrated their straightforward applications by synthesizing aminoglycoside lipidic derivative molecules that stimulate a potent immune response and enhance tumor regression in mice.

In this report, we first demonstrated the influence of naturally occurring cationic headgroup structure in immune stimulation by lipoaminoglycosides. The 4,6‐DDS ring subclass (DOST and DOSK) was able to trigger a strong upregulation of the cellular immune response compared to molecules from the 4,5‐DDS ring subclass (DOSP and DOSN), indicating a direct recognition of lipoaminoglycoside headgroups by immune‐related receptors. Moreover, unlike prior studies that have reported that immunomodulatory properties of ionizable lipids are due to the recognition of their dense positive charges as a danger signal,^11^, ^32^, ^33^, ^34^, ^35^ we did not observe any correlation between the number of positive charges and immune stimulation. Although DOSK possesses three positive charges and appeared to be the best adjuvant in this study, DOSN was not able to induce any immune stimulation, even though it has three additional positive charges.

Interestingly, the amphiphilic properties and integrity of lipoaminoglycosides are a prerequisite to confer immunomodulatory properties. Indeed, aminoglycosides alone without linkage to hydrophobic moiety were not able to induce immune stimulation. Further, change in the hydrophilic/hydrophobic balance of lipoaminoglycosides by modifying the spacer structure led to huge modification of their immunostimulatory properties. Indeed, using the shortest possible spacer (succinyl, DOST) allowed triggering the best cell stimulation, while increasing spacer size with two methylene units (adipyl, DOAT) dramatically hampered the stimulation efficiency. Additionally, it is known that the introduction of biodegradable functions within the spacer structure can influence the stability and integrity of lipids in intracellular or extracellular compartments.^36^, ^37^, ^38^ In the work described herein, we have demonstrated that destabilizing lipoaminoglycosides by adding a diester functional group (DOSST) also decreased their immunostimulatory properties. Moreover, engineering aminoglycoside lipidic derivatives with a disulfide bridge in the spacer led to inert molecules for cells. As this function can be degraded in intracellular compartments resulting in the separation of the headgroup and the hydrophobic segment,^36^, ^39^ this demonstrates that both the amphiphilic nature and the integrity of lipoaminoglycosides should not only be conserved in the extracellular compartment prior to cell interaction but also once the particles have been internalized by cells.

The nature of the hydrophobic segments also drastically impacted the immunostimulatory properties of cationic lipids. Aminoglycosides derivatives made with a cholesterol hydrophobic segment did not induce any significant cell stimulation, which is consistent with previous data from the literature demonstrating that cholesterol hydrophobic domains are less inflammatory than fatty acids hydrophobic domains.^40^ Regarding the composition of the alkyl chains, we demonstrated that cationic lipids with 2 oleyl chains (DOST, C18:1) better stimulated cell immunity compared to their saturated counterpart (DSST, C18:0).^41^ Unlike previous observations of cationic lipids' immunostimulatory properties,^27^ saturated alkyl chains with 14 (DMST) or 16 (DPST) carbons did not induce strong cell stimulation. Taken together those results suggest that the fluidity of the lipids once formulated in solution is an important parameter to consider.

Multiple strategies have been used to optimize lipidic‐derived immunomodulators to more efficiently target extracellular receptors. However, our results clearly demonstrated that the internalization mechanism of such lipids is a crucial parameter to induce a potent immune response. Although three main endocytosis pathways are generally used by cells to internalized molecules: clathrin‐ and caveolae‐mediated endocytosis and macropinocytosis,^32^, ^33^, ^42^ our results showed that caveolae‐dependent endocytosis was responsible for cell stimulation by our most potent lipoaminoglycosides DOSK. This is likely due to the formation of small vesicles, compatible with caveolae‐mediated internalization (20–100 nm). However, we cannot exclude a minor involvement of clathrin‐mediated endocytosis and macropinocytosis. Their inhibition also slightly impacted cytokine and chemokine expression, reflecting the presence of some DOSK vesicles above ≈100 nm in diameter as observed. Nevertheless, our data strongly suggest that DOSK and DOST lipoaminoglycosides are able to effectively stimulate immune gene expression via an intraendosomal or intracytoplasmic receptor.

Detection of ionizable lipids by immune‐related receptors has been described to happen mainly by the interaction between the hydrophobic segment of these molecules and TLR2 or TLR4.^26^, ^27^, ^28^ From a structural point of view, the best TLR2 ligands usually carry a hydrophobic C14:0 or C16:0 domain, while for TLR4 the best ligands possess a C12:0 or C14:0 domain. Although C14 saturated chains seem to be most adapted to activate TLR signaling pathways, our experiments show the opposite results as DMST (C14:0) was poorly immunogenic. More strikingly DOST and DOSK, both carrying a dioleyl hydrophobic segment (C18:1), induced a significant stimulation of the cell immune response in cells lacking TLR2 and TLR4. Thus, we hypothesize that another specific receptor is involved in lipoaminoglycoside recognition. This receptor seems to be of particular importance since we observed the potent activation of both innate immune pathways (NFkB and interferon regulatory factors). The ionizable lipids observed in this report are based on specific aminoglycoside headgroups, previously described as molecules triggering a specific allosteric activation of phosphatidylinositol phospholipase C enzyme (independent of their polycationic nature).^13^ While the grafting of aminoglycoside headgroups to hydrophobic segments via spacers could have altered this interaction, our results strongly support that PLC‐mediated signalization is the predominant signaling pathway involved in lipoaminoglycoside‐based innate immune stimulation. Indeed, inhibition of PLC by U73122 led to a dramatic decrease in cell stimulation by DOSK. This is also consistent with the endocytosis experiments, as PLC is present within the intracellular compartment.

Finally, lipoaminoglycosides have been tested for their potential as promising immunomodulators in vitro and in vivo to enhance complex immune reactions. DOSK's capability to enhance intragranulomatous human T cells stimulation and proliferation has validated our structural analysis, extending its potential therapeutic application in oncology. Intravenous injection of lipoaminoglycosides did not raise any safety concerns, as neither blood composition nor hematocrit levels were negatively influenced by DOSP and DOSK injection. Actually, DOSK injection even induced a slight increase in circulating monocytes, which is in accordance with several observations reporting the capacity of cationic liposomes to target monocytes specifically in whole blood over lymphocytes and granulocytes.^43^ The targeting and stimulation of monocytes by lipoaminoglycosides can facilitate their rapid migration to an inflamed site, their differentiation into macrophages or dendritic cells, and the induction of an efficient immune response.^34^, ^35^ Furthermore, we demonstrated that intratumoral injections of DOST and DOSK were able to reduce tumor growth, weight, and size significantly. Considering that we have already reported that lipoaminoglycosides used in this study induced no in vitro toxicity^16^, ^17^, ^18^, ^19^, ^20^ as well as no in vivo toxicity after intravenous injections, we strongly believe that DOST and DOSK antitumor effect is related to their intrinsic ability to stimulate local immune signaling pathways. This may alter the tumor immunosuppressive microenvironment, improving the antitumor activity of immune cells as well as their ability to infiltrate the tumor site. Nevertheless, as differences exist between preclinical and clinical models, further testing and optimizations will be implemented to confirm lipoaminoglycosides' therapeutic potential. For instance, injection of solutions in small tumors (200 mm^3^) can lead to their disruption, therefore breaking them in small aggregates and altering the tumor microenvironment. Also, the local concentration of adjuvants can have an impact on their efficacy. A too low concentration can promote immune tolerance while a too high concentration may kill the infiltrated immune cells leading to tumor progression.

## 4 Conclusion

This study successfully rationalized the design of polar head groups, spacers, and hydrophobic moieties to develop lipidic derivative molecules with optimal immunomodulatory properties. Indeed, we have determined that combining a polar aminoglycoside headgroup belonging to the 4,6‐DDS ring subclass, with a short spacer (succinyl), and a long unsaturated hydrophobic segment (dioleyl), allows a strong stimulation of the innate immune response via caveolae‐mediated endocytosis and PLC‐dependent signaling pathway. More broadly, lipoaminoglycosides can be used as immunomodulators to improve T cell stimulation and proliferation in vitro, but also in vivo leading to tumor growth reduction. Looking ahead, these lipoaminoglycosides might prove to be a versatile tool to stimulate antitumor immune cell activity, paving the way to combine these innovative adjuvants with current immunotherapies to enhance their immunogenicity and their therapeutic benefits.

## 5 Experimental Section

*Ionizable Lipids Preparation and Characterization*: Lipoaminoglycoside cationic lipids were kindly supplied by In‐Cell‐Art (Nantes, France), synthesized as previously described^14^, ^15^, ^16^, ^17^, ^18^ and stored as powders at −20 °C. Before the assays, they were diluted in endonuclease‐free water (2 mg mL^−1^ for in vitro studies; 40 mg mL^−1^ for in vivo studies) then sonicated for 10–15 min at room temperature. Stock solutions were stored up to 1 month at 4 °C.

The size distribution of lipoaminoglycosides formulated in solution was evaluated by DLS analysis (at an angle of 90°) and CryoEM. For DLS, lipoaminoglycoside vesicles were prepared by diluting each compound in complete DMEM medium (10 µg mL^−1^), and their size was measured using a laser light scattering apparatus (Autosizer 4700; Malvern Instruments, Orsay, France). The mean particle diameter was determined by multimodal fit analysis. For CryoEM, lipoaminoglycosides were diluted in complete DMEM (125 µg mL^−1^) and deposed on lacey carbon copper grids that were beforehand submitted to a standard glow discharge procedure. Grids were flash‐frozen into a liquid ethane bath using EM GP (Leica). Specimens were observed under low‐dose conditions using a cryo holder (Gatan, USA). Observations were performed with an FEI Tecnai F20 electron microscope operating at 200 kV, and images were acquired using a digital 2k × 2k USC1000 camera (Gatan).

*Cell and Culture Conditions*: Mouse skeletal muscle cells (C2C12; CRL1772, ATCC, Rockville, MD, USA) were cultured in DMEM supplemented with 10% heat‐inactivated fetal calf serum (FCS, Eurobio, Courtaboeuf, France), penicillin (100 U mL^−1^) (Life Technologies, Villebon sur Yvette, France), streptomycin (100 µg mL^−1^) (Life Technologies), and l‐glutamine (2 × 10^−3^ m) (Life Technologies).

MEF cells (CRL‐2907, ATCC) were maintained in IMDM supplemented with 10% heat‐inactivated FCS, penicillin (100 U mL^−1^), streptomycin (100 µg mL^−1^), and 1× nonessential amino acids (Life Technologies).

Immature dendritic cells (JAWSII; CRL‐11904, ATCC) were grown in RPMI1640 supplemented with 10% heat‐inactivated FCS, penicillin(100 U mL^−1^), streptomycin (100 µg mL^−1^), and murine GM‐CSF (5 ng mL^−1^).

Human embryonic kidney 293 cells (HEK293) TLR2/4‐negative (null) or expressing either human TLR2 (hTLR2) or human TLR4 (hTLR4) (Invivogen, Toulouse, France) were cultured in DMEM supplemented with 10% heat‐inactivated FCS, penicillin (50 U mL^−1^), streptomycin (50 µg mL^−1^), and normocin (100 µg mL^−1^) (Life Technologies).

Peritoneal cavity cells were obtained as described by Ray and Dittel^44^ and were cultured in RPMI 1640 supplemented with 10% FCS, penicillin (100 U mL^−1^), streptomycin (100 µg mL^−1^), and l‐glutamine (2 × 10^−3^ m).

BMDCs were extracted from femur bone marrow as described by Lutz et al.^45^ cultured during 10 days in 60 mm dish in order to reduced granulocytes contamination in RPMI 1640 supplemented with 10% FCS, penicillin (100 U mL^−1^), streptomycin (100 µg mL^−1^), l‐glutamine (2 × 10^−3^ m), 2‐mercaptoethanol (50 × 10^−6^ m), and murine GM‐CSF (30–200 U mL^−1^).

All cells were routinely maintained at 37 °C in a humidified 5% CO_2_/95% air containing atmosphere. The day before treatment, cells were seeded in 24‐well plates to achieve a confluence of 70–80%.

*In Vitro Stimulation*: The day after seeding, cell supernatants were replaced by fresh growth medium. Then, cells were treated for 2, 6, or 24 h with Lipoaminoglycosides (3–10 µg mL^−1^), ultrapure LPS (100 ng mL^−1^), or Pam3CSK4 (100 ng mL^−1^) (Invivogen) diluted in Opti‐MEM. When inhibitors of endocytosis or PLC were used, C2C12 cells were incubated for 1 h with 5‐(*N*‐ethyl‐*N*‐isopropyl)amiloride (150 × 10^−9^ m), chlorpromazine hydrochloride (9 × 10^−6^ m), genistein (90 × 10^−6^ m), or U73122 (1 × 10^−6^ m, Sigma‐Aldrich) before the addition of activators (Sigma‐Aldrich, Darmstadt, Germany). To confirm endocytosis inhibition efficiency, cells were treated with or without inhibitors and assessed by fluorescence microscopy for their ability to internalize FITC‐dextran 70k (Sigma‐Aldrich), FITC‐transferrin (Thermofisher), or bodipy‐LacCer (Thermofisher).

For the intragranuloma T cells proliferation study, human PBMCs were first labeled for 15 min at 37 °C in the dark with violet proliferation dye 450 (VPD, 1 × 10^−6^ m) (BD Bioscience) in PBS, then washed twice with PBS. 1 × 10^5^ VPD‐PBMCs were seeded in 96‐well round‐bottom plates (Falcon) and cultured with mycobacterium bovis (bacille calmette‐guerin, BCG) at an MOI of 1 in presence or absence of DOSK or DOSP (10 µg mL^−1^) in complete medium RPMI 1640 (200 µL) supplemented with l‐glutamine (2 × 10^−3^ m), penicillin–streptomycin (10 mg mL^−1^, Gibco), and 8% of human serum (local production). After 7 days of incubation at 37 °C in 5% CO_2,_ the proliferation of target T cells was assessed by flow cytometry analysis of VPD dilution.

*Cell Viability*: Twenty‐four hours after treatment, cytotoxicity was measured using the Cell Titer 96 nonradioactive cell proliferation assay according to manufacturer's instructions (Promega, Lyon, France). After solubilization of the formazan crystals, optical density was measured at 560 nm using a Victor X‐3 multilabel plate reader (Perkinelmer, Villebon sur Yvette, France). Cell survival in the treated samples was calculated using a regression curve established from untreated standard serial dilutions of the cell suspension.

*Measurement of the Immune Response*: After stimulation, cell culture supernatants were collected and assayed for mouse CXCL10 secretion by using DuoSet Mouse CXCL10/IP‐10/CRG‐2 ELISA kit (R&D Systems, Minneapolis, USA) according to the manufacturer's instructions, using a Victor X3 (Perkinelmer) microplate reader.

IFNβ1, IL‐6, CXCL10, DAI, and IFI204/16 expression was determined by RT‐qPCR. Total RNA from cells was extracted using the nucleospin RNA/protein kit (Macherey‐Nagel, Hoerdt, France), according to the manufacturer protocol. RT‐qPCR was then realized in two phases: first, reverse transcription of RNA to cDNA was conducted using high capacity cDNA reverse transcription kits (Life Technologies) on GeneAmp PCR System 9700 Thermal Cycler (Life Technologies). Then, real‐time PCR was effectuated using Taqman Gene expression assays on StepOnePlus real‐time PCR system (Life Technologies).

Probes used to amplify specific gene products from murine cDNA (Life Technologies) were: for IL‐1β: Mm00434228_m1; for IL‐6: Mm00446190_m1; for CXCL10: Mm00445235_m1; for DAI: Mm00457979_m1; for IFI204: Mm00492602_m1; for HPRT (housekeeping gene): Mm00446966_m1. Probes used to amplify specific gene products from human cDNA (Life Technologies) were: for IL‐1β: Hs01077958_s1; for IL‐6: Hs00985639_m1; for CXCL10: Hs01124251_g1; for DAI: Hs01090106_m1; for IFI16: Hs00194261; for HPRT (housekeeping gene): Hs99999909_m1.

DAI and IFI204 productions were confirmed by Western Blotting assay. Whole cell proteins were extracted using the nucleospin RNA/protein kit (Macherey‐Nagel), fractionated by SDS‐PAGE on miniprotean TX gel 4–20%, and transferred to a 0.2 µm nitrocellulose trans‐blot turbo membrane (Bio‐Rad, Marnes‐la‐Coquette, France) according to the manufacturer's instructions. The membranes were blocked with 5% nonfat milk in tris‐buffered saline for 1 h and then incubated overnight with primary antibody at 4 °C. After washing membranes three times with tris‐buffered saline, they were incubated with horseradish peroxidase‐conjugated secondary antibody for 1 h at room temperature. The peroxidase activity associated with protein bands was detected by enhanced chemiluminescence using Clarity Western ECL blotting substrate (Bio‐Rad) and measured with a ChemiDoc MP System (Bio‐Rad). Antibodies used to detect murine protein bands were: for DAI: sc‐271483 (Santa Cruz Biotechnology, Dallas, USA); for IFI204: ab104409 (Abcam, Paris, France); for anti‐rabbit‐HRP: sc‐2054 (Santa Cruz Biotechnology); for anti‐mouse‐HRP: sc‐2055 (Santa Cruz Biotechnology).

*Animals*: Eight‐week old BALB/c mice or Swiss mice (Elevage Janvier, Le Genest, France) were housed in conventional conditions according to INSERM (Institut National de la Santé et de la Recherche Médicale) guidelines. All animal experiments were performed according to the recommendations of French Ministry of Higher Education and Research and approved by an ethics committee on animal experimentation under reference APAFIS#7897 and 4118.

*In Vivo Study*: For blood formulation and hematocrit level measurement, Swiss mice were injected intravenously with DOSP or DOSK (100 µg). Blood samples were collected from 1 day before to 3 days after injection, and analysis with Hemavet 950FS (Drew Scientific, Dallas, USA).

Breast cancer tumors were established in BALB/c mice by intramammary injection of 1 × 10^5^ 4T1‐luciferase cells. Ten days after cells injection (median tumor size = 100–200 mm^3^), mice were treated with two weekly intratumoral injections as follows: saline solution (control), DOSP (1 mg), DOST (1 mg), and DOSK (1 mg). Then, at day 19 after initiation of tumor growth, mice were sacrificed, and tumors were excised for weight and size analysis.

*Statistical Analysis*: Statistical analyses were performed using GraphPad Prism software (La Jolla, CA, USA). Values are shown as mean ± SEM. Data were analyzed using Mann‐Whitney test, *p < 0.05, **p < 0.01, and ***p < 0.01.

## Conflict of Interest

B.P. owns stock in In‐Cell‐Art, which commercializes lipidic aminoglycodise derivatives.

## Supporting information

Supplementary
