# Supplementary material for: Lipidic Aminoglycoside Derivatives: A New Class of Immunomodulators Inducing a Potent Innate Immune Stimulation
Source: Adv Sci (Weinh). 2019 Jul 15;6(16):1900288. doi: 10.1002/advs.201900288 (PMC6702646; doi:10.1002/advs.201900288)
Supplement: Supplementary file 1 — Supplementary [file ADVS-6-1900288-s001.pdf]

Copyright WILEY-VCH Verlag GmbH & Co. KGaA, 69469 Weinheim, Germany, 2019.

ADVANCED SCIENCE
Open Access

Supporting Information

for *Adv. Sci.*, DOI: 10.1002/advs.201900288

Lipidic Aminoglycoside Derivatives: A New Class of Immunomodulators Inducing a Potent Innate Immune Stimulation

*Thibault Colombani, Thomas Haudebourg, Marion Decossas, Olivier Lambert, Grace Ada Da Silva, Frederic Altare, and Bruno Pitard**

WILEY-VCH

Copyright WILEY-VCH Verlag GmbH & Co. KGaA, 69469 Weinheim, Germany, 2019.

Supporting Information

Lipidic Aminoglycoside Derivatives: a New Class of Immunomodulators Inducing a Potent Innate Immune Stimulation.

*Thibault Colombani, Thomas Haudebourg, Marion Decossas, Olivier Lambert, Grace Ada Da Silva1, Frederic Altare, and Bruno Pitard.**

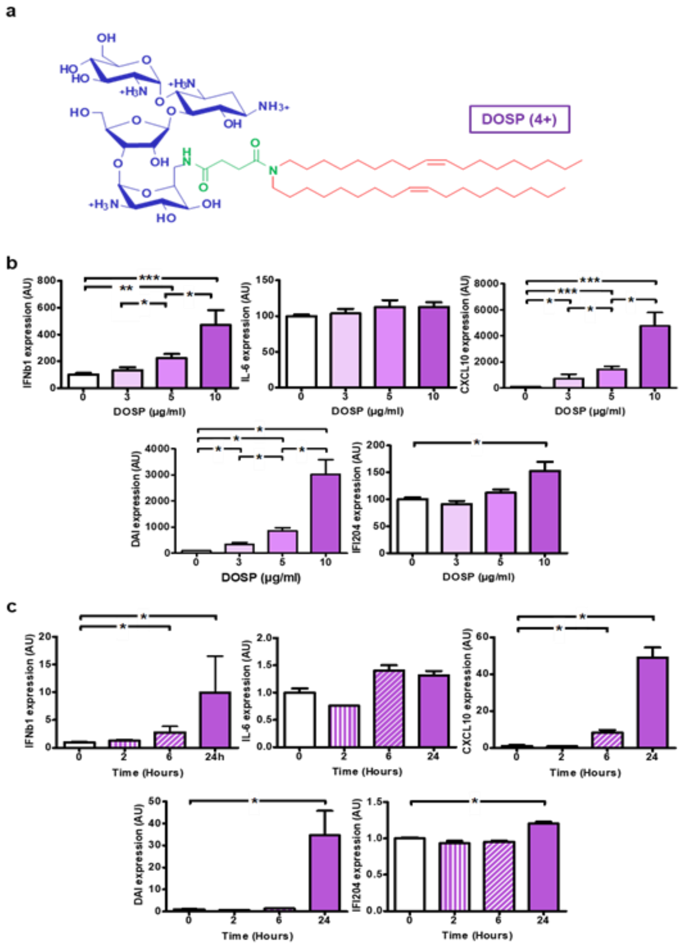

Figure S1. Effect of DOSP on cell immune response. (**a**) Structure of Di-Oleyl-Succinyl-Paromomycin (DOSP). (**b-c**) Effect of DOSP on the expression of innate immune genes as a function of (**b**) the concentration or (**c**) the duration of treatment. (**b**) Mouse myoblast cells (n=6) were incubated for 24 hours with DOSP at a concentration of 0 to 10 µg/mL. (**c**) Mouse myoblast cells (n=4) were not incubated or incubated for 2, 6 or 24 hours with DOSP at a concentration of 10 µg/mL. Cytokine (IFNβ1 and IL-6), chemokine (CXCL10) and DNA sensor (DAI and IFI204) expression levels in cells were determined by RT-qPCR analysis, normalized against the expression levels of HPRT (housekeeping gene) and compared to non-treated cells (AU=1). Data are expressed as the $2^{-\Delta\Delta}$CT formula and are shown as mean ± SEM. Values are shown as mean ± SEM. Data were analyzed using Mann-Whitney test, *p<0.05, **p<0.01, and ***p<0.01.

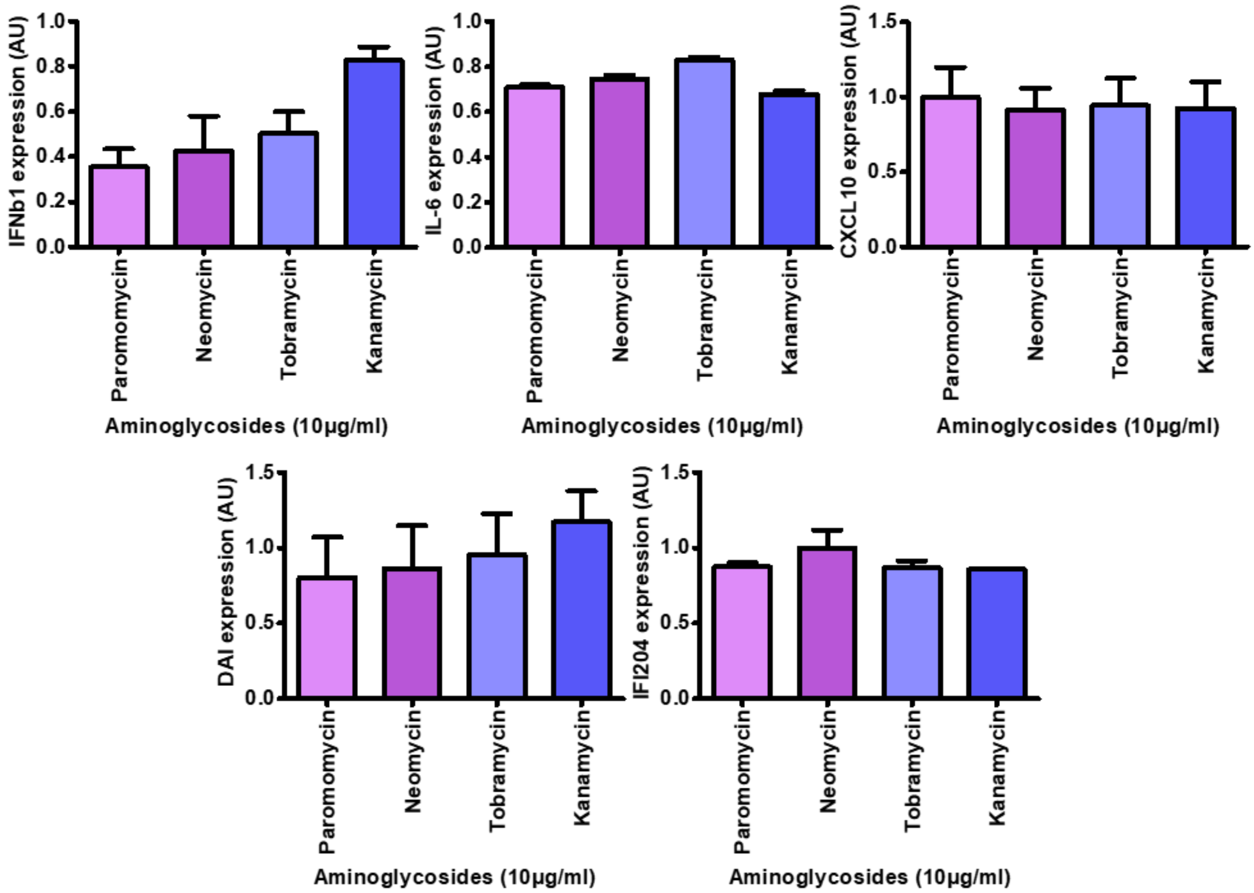

Figure S2. Effect of aminoglycosides alone on cell immunity. Induction of innate immune gene expression by aminoglycosides in C2C12 cells. Mouse myoblast cells (n=4) were incubated for 24 hours with 10µg/mL of paromomycin, neomycin B, tobramycin or kanamycin A belonging to the 4,5-disubstituted 2-deoxystreptamine ring subclass or 4,6-disubstituted 2-deoxystreptamine ring subclass respectively. Cytokine (IFNβ1 and IL-6), chemokine (CXCL10) and DNA sensor (DAI and IFI204) expression levels in cells were determined by RT-qPCR analysis, normalized against the expression levels of HPRT (housekeeping gene) and compared to non-treated cells (AU=1). Values are shown as mean ± SEM. Data were analyzed using Mann-Whitney test, *p<0.05.

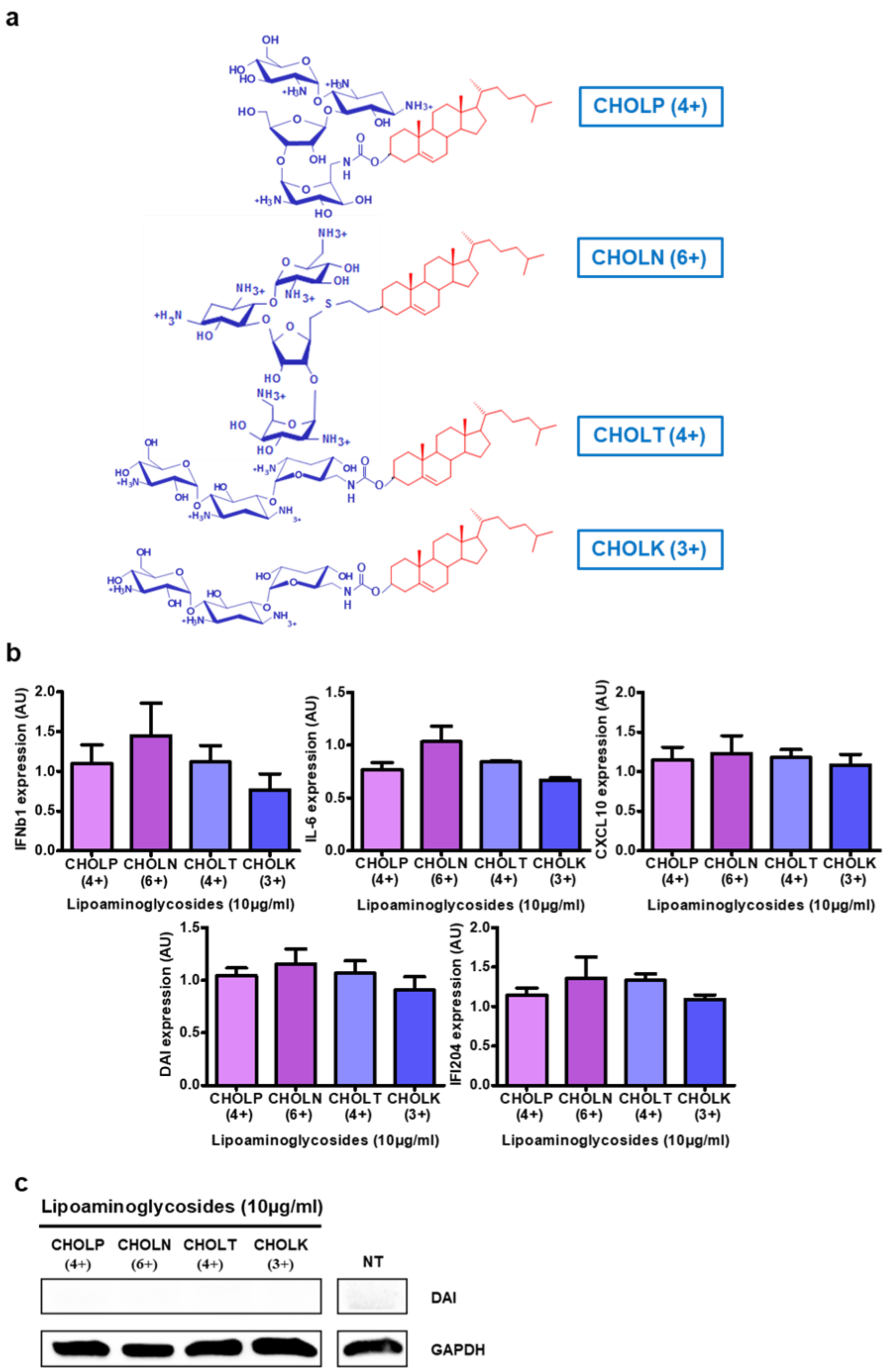

Figure S3. Cholesterol-derived aminoglycosides failed to induce cell stimulation. (**a**) Structure of Cholesterol derived cationic lipids: CHOLT (Cholesterol-Tobramycin), CHOLK (Cholesterol-Kanamycin A), CHOLP (Cholesterol-Paromomycin) and CHOLN (Cholesterol-Neomycin B). (**b-c**) Stimulation of innate immune response by Cholesterol derived cationic lipids in C2C12 cells. Mouse myoblast cells (n=4) were incubated for 24 hours with 10µg/mL of CHOLP, CHOLN, CHOLT or CHOLK, belonging to the 4,5-disubstituted 2-deoxystreptamine ring subclass or 4,6-disubstituted 2-deoxystreptamine ring subclass

respectively. (**b**) Cytokine (IFNβ1 and IL-6), chemokine (CXCL10) and DNA sensor (DAI and IFI204) expression levels in cells were determined by RT-qPCR analysis, normalized against the expression levels of HPRT (housekeeping gene) and compared to non-treated cells (AU=1). Data are expressed as the $2^{-\Delta\Delta}$CT formula. (**c**) DAI DNA sensor proteins contained into cells were determined by Western Blot analysis and the level of GAPDH was used as a protein loading control. Values are shown as mean ± SEM. Data were analyzed using Mann-Whitney test, *p<0.05.

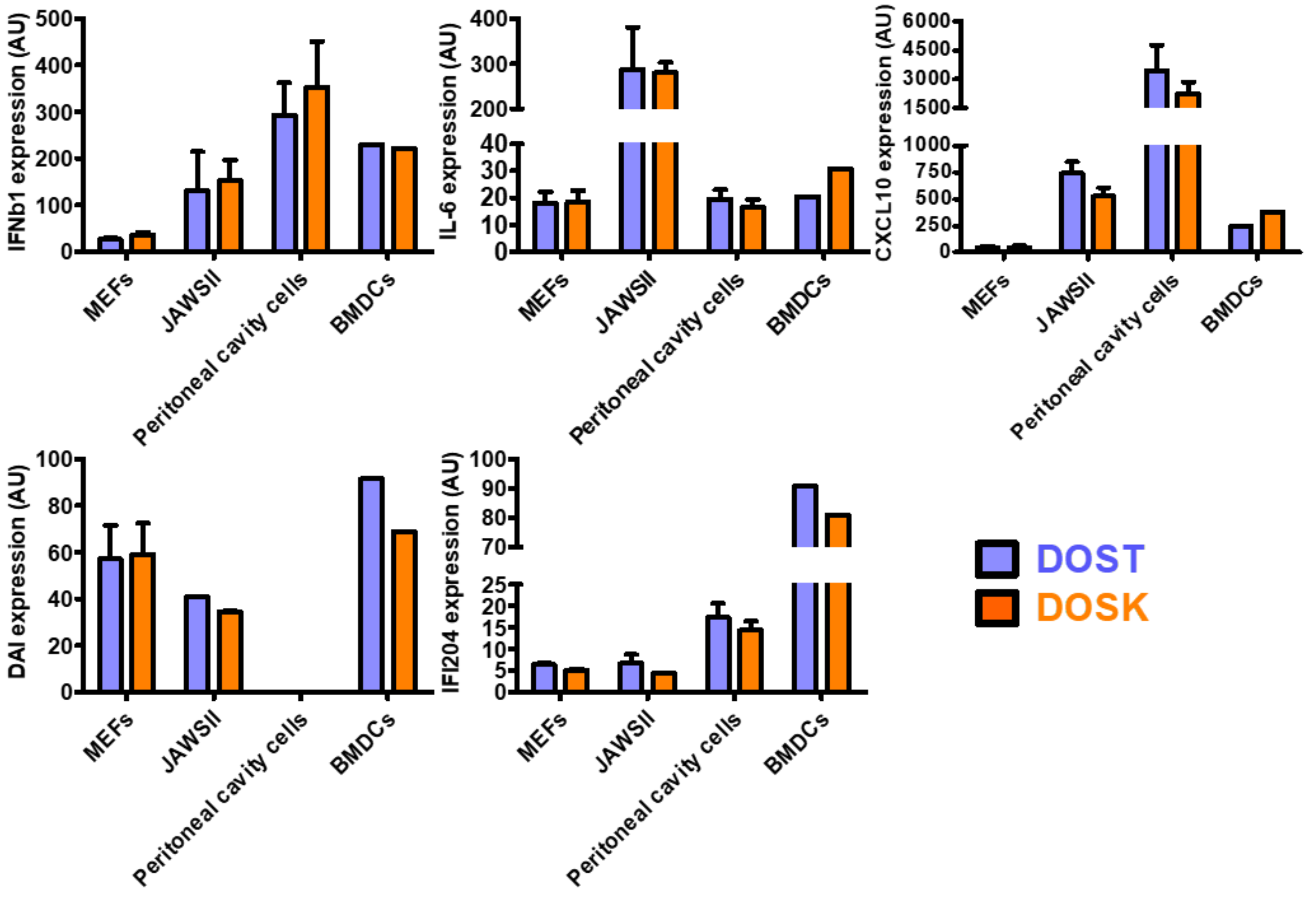

Figure S4. DOST and DOSK activate innate immune response on a wide panel of cells. MEFs, JAWSII, peritoneal cavity cells and BMDCs (n=4) were incubated for 24 hours with DOST and DOSK at a concentration of 10 µg/mL. Cytokine (IFNβ1 and IL-6), chemokine (CXCL10) and DNA sensor (DAI and IFI204) expression levels into cells were determined by RT-qPCR analysis, normalized against the expression levels of HPRT (housekeeping gene) and compared to non-treated cells (AU=1). Data are expressed as the $2^{-\Delta\Delta}$CT formula and are shown as mean ± SEM.

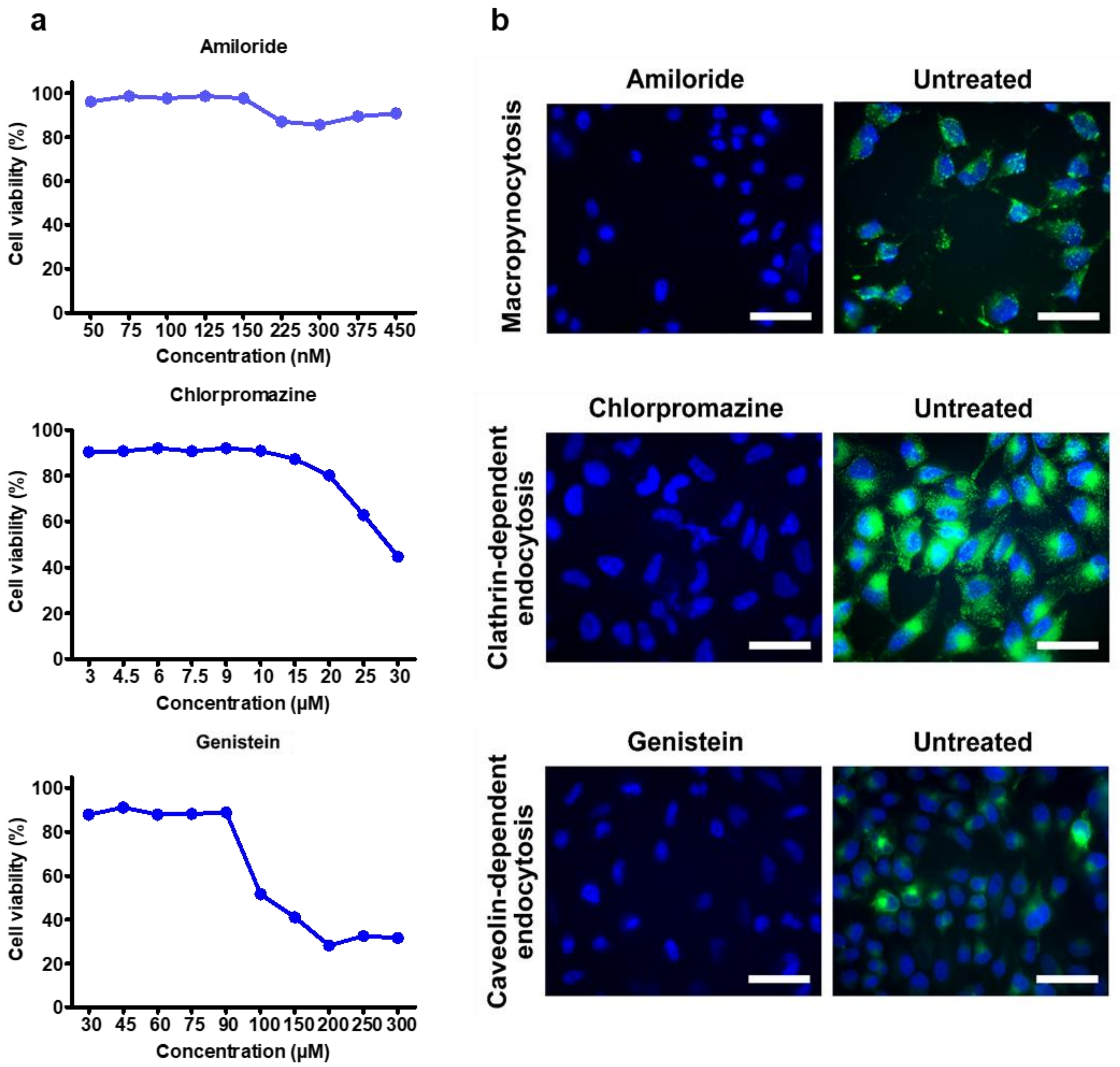

Figure S5. Validation of endocytosis inhibitors efficacy. (**a**) Percentage cell survival of C2C12 cells. Mouse myoblast cells (n=4) were treated with various concentration of amiloride (50 to 450 nM), chlorpromazine (3 to 30 µM), and genistein (30 to 300 µM) for 24 hours. Cell viability was evaluated using the Cell Titer 96 Non-Radioactive Cell Proliferation Assay. (**b**) Endocytosis inhibition by fluorescence microscopy. Macropinocytosis inhibition was confirmed by monitoring FITC-Dextran internalization by C2C12 cells in presence or

absence of 150nM of amiloride. Clathrin-dependent endocytosis inhibition was confirmed by monitoring FITC-transferrin internalization by C2C12 cells in the presence or absence of 9µM of chlorpromazine. Caveolin-dependent endocytosis inhibition was confirmed by monitoring bodipy-LacCer internalization by C2C12 cells in presence or absence of 90µM of Genistein. Blue = DAPI staining. Pictures are representative of n=5 samples. Scale bare = 25µm.

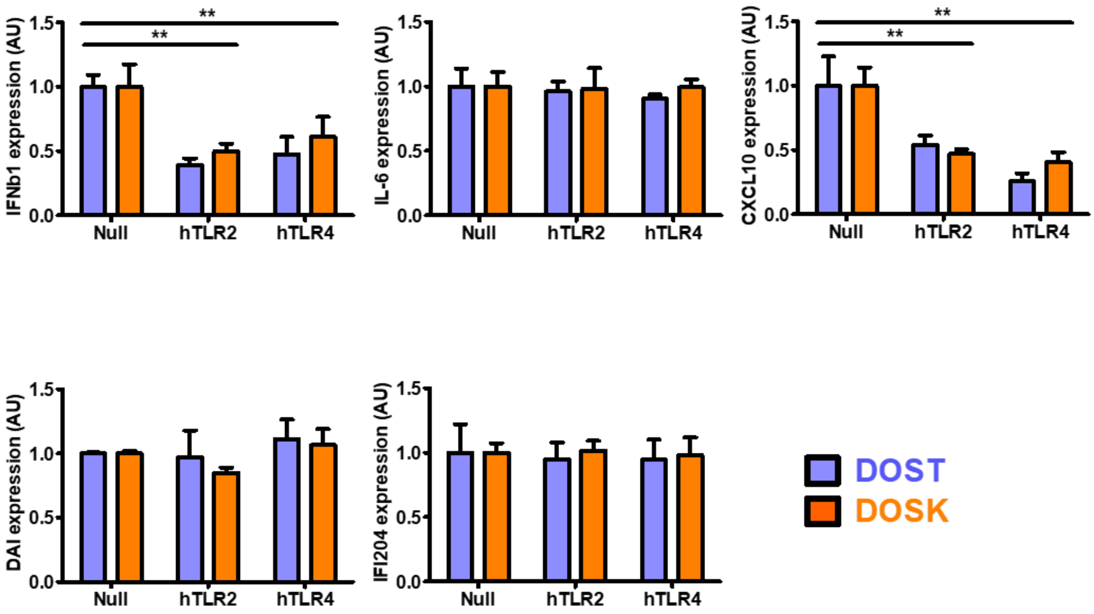

Figure S6. TLR2 and 4 decrease DOST and DOSK immunostimulatory properties. TLR2/4-negative HEK293 cells (null), or HEK293 cells expressing human TLR2 (hTLR2) or TLR4 (hTLR4) (n=4) were incubated for 24 hours with 10µg/ml of DOST (blue) or DOSK (orange). Cytokine (IFNβ1 and IL-6), chemokine (CXCL10) and DNA sensor (DAI and IFI204) expression levels in cells were determined by RT-qPCR analysis, normalized against the expression levels of HPRT (housekeeping gene) and compared to TLR2/4-negative HEK293 cells (null) (AU=1). Values are shown as mean ± SEM. Data were analyzed using Mann-Whitney test, *p<0.05, **p<0.01.
